# Supplementary material for: The effects of dietary iron supplementation on bacterial infections in Manduca sexta larval hemolymph
Source: PLoS One. 2026 Aug 14;21(8):e0346464. doi: 10.1371/journal.pone.0346464 (PMC13475885; doi:10.1371/journal.pone.0346464)
Supplement: Supplementary file 1 [file pone.0346464.s001.pdf]

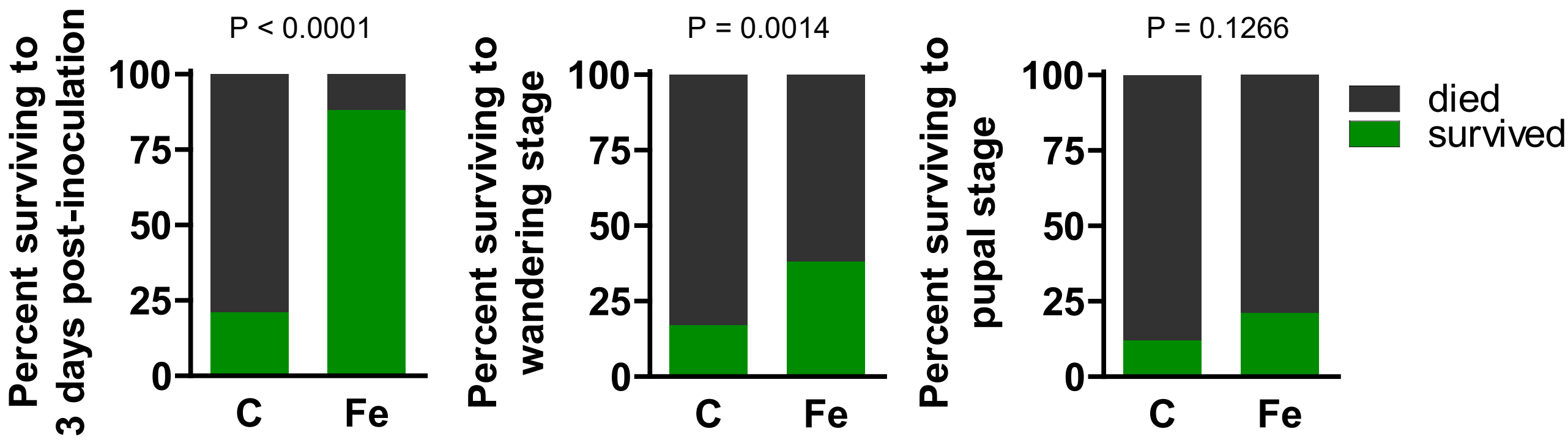
P < 0.0001
Percent surviving to 3 days post-inoculation
100
75
50
25
0
C
Fe
P = 0.0014
Percent surviving to wandering stage
100
75
50
25
0
C
Fe
P = 0.1266
Percent surviving to pupal stage
100
75
50
25
0
C
Fe
died
survived
